# Traffic flow digital twin generation for highway scenario based on radar-camera paired fusion

**DOI:** 10.1038/s41598-023-27696-z

**Published:** 2023-01-12

**Authors:** Yanbing Li, Weichuan Zhang

**Affiliations:** 1 School of Electronic and Information Engineering, Beijing Jiaotong University, Beijing, 100044 China; 2 Institute for Integrated and Intelligent Systems, Griffith University, Brisbane , QLD Australia

**Keywords:** Engineering, Electrical and electronic engineering

## Abstract

Autonomous driving is gradually moving from single-vehicle intelligence to internet of vehicles, where traffic participants can share the traffic flow information perceived by each other. When the sensing technology is combined with the internet of vehicles, a sensor network all over the road can provide a large-scale of traffic flow data, thus providing a basis for building a traffic digital twin model. The digital twin can enable the traffic system not only to use past and present information, but also to predict traffic conditions, providing more effective optimization for autonomous driving and intelligent transportation, so as to make long-term rational planning of the overall traffic state and enhance the level of traffic intelligence. The current mainstream traffic sensors, namely radar and camera, have their own advantages, and the fusion of these two sensors can provide more accurate traffic flow data for the generation of digital twin model. In this paper, an end-to-end digital twin system implementation approach is proposed for highway scenarios. Starting from a paired radar-camera sensing system, a single-site radar-camera fusion framework is proposed, and then using the definition of a unified coordinate system, the traffic flow data between multiple sites is combined to form a dynamic real-time traffic flow digital twin model. The effectiveness of the digital twin building is verified based on the real-world traffic data.

## Introduction

With the progress of computing power and communication technology, real-time environmental perception and path planning can be realized in recent years, which promotes the rapid development of autonomous driving technology. An important research direction of autonomous driving is the realization of single-vehicle self-driving. However, the perception capability of an individual vehicle is limited to its surroundings, which has limited benefits for improving the operational efficiency of large-scale traffic. In order to achieve efficient autonomous driving, current trend is to connect vehicles to each other as well as to connect vehicles and road facilities^1,2^. By sharing traffic flow information, vehicle motion and road traffic control can be jointly optimized for improving overall traffic efficiency^3,4^. This is the concept of the internet of vehicles (IoV)^5^.

As an important application of internet of things (IoT) technology in intelligent transportation, IoV enables the task of environmental sensing to be accomplished not only by sensors on vehicles but also by roadside sensing devices^6^. When the IoV is formed, for a vehicle on the road, it can get the traffic condition of a broader area, e.g., a city, at a certain moment, which is useful for long-term driving planning. In addition, when the traffic flow state of an area is obtained, the perceived traffic flow data can be utilized for generating a traffic digital twin (DT) model. Based on the DT model, the traffic flow state at future moments can be predicted^7^, which will provide more knowledge for autonomous driving of vehicles and traffic light control of roads^8,9^. Therefore, it will greatly enhance the function of intelligent transportation in cities^10^.

In IoV solutions for autonomous driving, the large amount of traffic flow information is updated in real time, which leads to a big challenge for computational systems since it must complete information collection and processing in a very short time and provide decision guidance for autonomous vehicles. With the help of DT technology, intelligent transportation systems have the potential to solve such challenges.

A typical autonomous driving application is the trajectory prediction of traffic participants. For an autonomous vehicle, its behavior needs to depend on the trajectory prediction results of its surrounding traffic participants. Although existing machine learning techniques, such as deep neural networks, provide excellent prediction approaches, model updating is a key issue in practical applications. DT model is precisely an effective way to address online model updates. A DT model of surrounding vehicles is built for providing real-time information input to a long short term memory (LSTM) neural network in order to keep the network dynamically updated and achieve real-time prediction of surrounding vehicle trajectories^7^. This study shows us the key role played by the DT model in making decisions for individual autonomous vehicles. It is a local real-time replica of the physical world that can provide the necessary digital information for individual autonomous driving decisions.

DT model on a larger scale is used to analyze and predict the state of traffic flow at the city level, which provides traffic control decisions for city managers to alleviate road congestion^8^. Based on the traffic flow information collected by multiple sensors, city-level traffic flow DT models are built to accurately predict the traffic flow status on city roads even if data is missing in some areas.

The above studies discuss the important role played by DT technology in the development of autonomous driving and intelligent transportation. However, the generation of DT models is a prerequisite for their application. The accuracy of the DT model can directly affect the effectiveness of subsequent applications. Therefore, it is important to establish an effective DT model based on sensors. Currently, traffic flow information acquisition by roadside sensors mainly relies on cameras^11^. With the development of radar technology, more and more millimeter wave (mmWave) radars have been used for roadside sensing^12^. It is well known that radar has good radial distance and speed measurement accuracy as well as all-weather working capability, these features make radars and cameras work well together^13^. Combining the features of radar and camera sensing and fusing the information of the two sensors can provide more accurate traffic flow information for building traffic DT model.

In terms of sensor fusion purpose, radar camera fusion can be mainly divided into target detection and recognition oriented fusion and target tracking oriented fusion. In the target detection and recognition oriented fusion, the consideration is how to improve the detection or recognition accuracy^14–17^. For instance, a radar and camera fusion framework is proposed, where the camera is used for more accurate detection in the region of interest provided by the radar, thus effectively reducing false alarms from the radar detections^15^. Target classification based on radar and camera fusion for roadside application is studied^16^, in which enhanced evidence theory is employed for belief assignment to solve target classification in extreme light conditions.

The above methods are able to obtain high target detection and recognition performance, but for traffic DT scenarios, accurate target location information is required for subsequent motion prediction and state evolution. Therefore, target tracking oriented fusion is more appropriate for DT applications. There have been some tracking oriented sensor fusion studies^18–21^, in which the improvement of target tracking accuracy have been discussed. In these studies, two strategies are usually used for the acquisition of target fusion trajectories. The first is track-to-track fusion, in which the radar and camera track the target separately to form their respective trajectories, and then inter-sensor fusion is performed based on the sensor trajectory output. The second is detection-to-detection fusion, in which the radar and camera do not track the target, but input the detection results into a fusion filter, which directly outputs the fused target trajectory.

These fusion methods play an active role in improving the sensing capability of single vehicle or intelligent traffic systems. For the high accuracy acquisition of traffic flow information in IoV applications, this paper considers a DT model generation approach based on roadside radar and camera sensor fusion in highway scenarios. The vehicles are tracked in real time by using radar-camera pairs distributed at multiple roadside sites and a DT model of traffic flow on the road is formed. Combining the respective advantages of radar and camera, the DT model constructed by sensor fusion has better location accuracy and robustness to light and weather conditions, which can provide reliable traffic flow information for subsequent smart traffic applications. The main contributions of this paper are as follows.

1. An end-to-end generation approach from raw sensory data to a highway DT model is proposed. Based on pairs of radar camera sensors, vehicles are tracked to form a DT model of highway traffic flow, which provides information for subsequent traffic optimization.
2. A novel road feature-based radar camera calibration method is proposed. The mounting errors of the radar and camera are automatically calibrated using intermediate belt features on the highway. The proposed method aligns the two sensors in space without the support of additional equipment.
3. Combining the measurement error distribution characteristics of radar and camera, a Kalman filter framework-based sensor data fusion method is proposed.

The rest of the paper is organized as follows: first, radar and camera models for traffic flow sensing is introduced, then the DT model generation approach is presented. Finally, the effectiveness of sensor calibration based on scene-feature and highway DT model generation based on sensor fusion is verified by real-world scenario experiment.

## Sensor model for DT generation

### Radar model

The most advanced mmWave radar sensors utilize frequency modulated continuous wave (FMCW) technology, where chirp sequence modulation with stretching processing is usually used for transmitted waveform and received processing. Meanwhile, multiple-input multiple-output (MIMO) structure are employed for increasing the effective antenna array aperture and number^22,23^. MmWave radar detects targets by emitting a set of chirp sequence as follows.

1 $$\begin{aligned} \begin{aligned} & s_{t}(t)=\sum _{m=1}^{M} \Lambda _{t} W_{T}(t-t_{s}) \sin \left[ 2 \pi \left( f_{c}+\frac{1}{2} \gamma t_{f}\right) t_{f}\right] ,\\&\text {with } W_{T}(t)=\left\{ \begin{array}{ll} 1 &{} 0\le t \le T \\ 0 &{} \text {otherwise} \end{array}\right. , \end{aligned} \end{aligned}$$

where *M* is the total number of chirps in the sequence, with pulse repetition time (PRT) of chirps $T_{r}$, $t_{s}=(m-1)T_{r}$, is the slow-time, which is used to measure time change over multiple RPTs, $t_{f}$ denotes the fast-time, which is used to measure time change in a single chirp, $t=t_{s}+t_{f}$, is the total time, $\Lambda _{t}$ is the chirp amplitude, $f_{c}$ is the carrier frequency, with the chirp sweep bandwidth *B* and the chirp time width *T*, $\gamma =B/T$ is the chirp rate.

When the transmitted chirp sequence meets a target, the sequence will be scattered by the target, and return to the radar after a delay caused by the propagation of the transmitted signal in free space. Then the echo signal is received by the radar and mixed with the transmitted signal in the receiver for obtaining the beat-frequency signal in terms of Eq. (1). The beat-frequency signal is^22^

2 $$\begin{aligned} \begin{aligned} s_{r}(t) = \sum _{m=1}^{M} \Lambda _{r}&W_{T}(t-\tau -t_{s}) \exp \left[ 2 \pi \left( f_{d} t_{s}-f_{b} t_{f} \right) \right] ,\\ \text {with }&\tau =\frac{2(R+v_{r} t)}{c} ,\\&f_{b}=\gamma \tau ,\\&f_{d}=\frac{2v_{r}}{\lambda }, \end{aligned} \end{aligned}$$

where *R* and $v_{r}$ are the radial distance and the velocity of the target respectively, *c* is the speed of light, $\tau$ represents the delay between the transmitted and the received signals, $f_{b}$ is the beat frequency, $f_{d}$ is the Doppler frequency, and $\lambda$ is the wavelength of the transmitted signal.

It is worth to note that the above analysis is for one receiver antenna channel. When the radar has receiver antenna array with *N* elements, each antenna element will receive chirp sequences independently. By using the first antenna array element as a reference, and assuming that the antenna array element interval is *d* and the azimuth angle of the target relative to the radar antenna normal is $\theta$, the target echo received by the antenna array element can be expressed as^22^

3 $$\begin{aligned} s_{r a}(t)=\left[ \begin{array}{c} s_{r 1} \\ \vdots \\ s_{r n} \\ \vdots \\ s_{r N} \end{array}\right] =\left[ \begin{array}{c} s_{r}(t) \\ \vdots \\ s_{r}(t) \exp \left( 2 \pi \frac{(n-1) d \sin \theta }{\lambda }\right) \\ \vdots \\ s_{r}(t) \exp \left( 2 \pi \frac{(N-1) d \sin \theta }{\lambda }\right) \end{array}\right] . \end{aligned}$$

**Figure 1:**
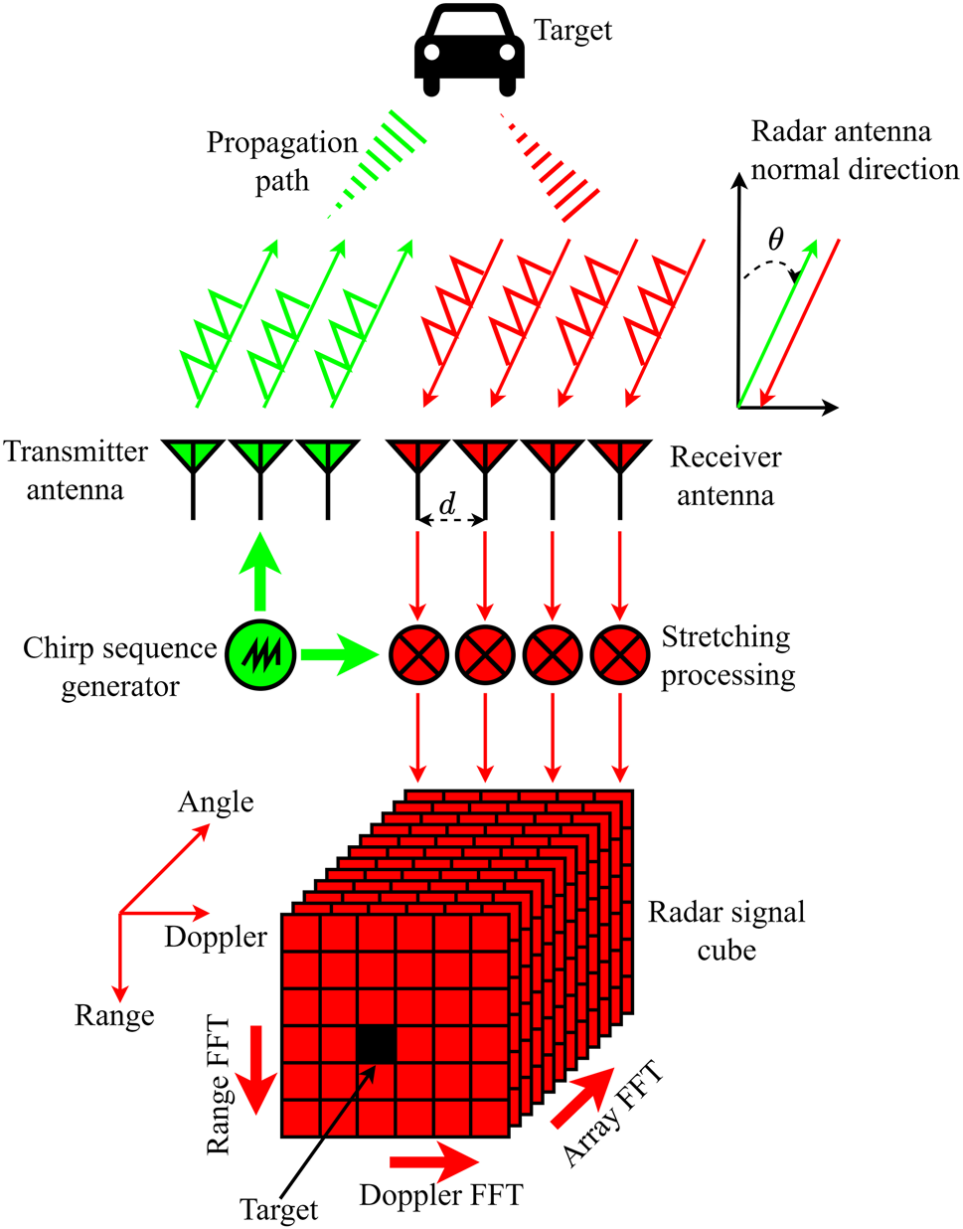
Signal processing flowchart of chirp sequence modulation and MIMO structure.

A typical mmWave radar signal processing flow is shown in Fig. 1. The transmitter antenna array emits chirp sequence, then the chirp sequence interacts with the target and returns to the receiver antenna array, followed by a stretching process for obtaining the radar signal cube. In this case, the target range and velocity can be obtained by a constant false-alarm rate (CFAR) detector after applying range FFT and Doppler FFT, aka range-Doppler processing, to the beat-frequency signal in terms of Eq. (2), and the target azimuth angle can be obtained by beamforming which is realized by the array FFT^22^. After the radar signal processing stage, the target ground plane position expressed in polar coordinates can be obtained.

### Camera model

**Figure 2:**
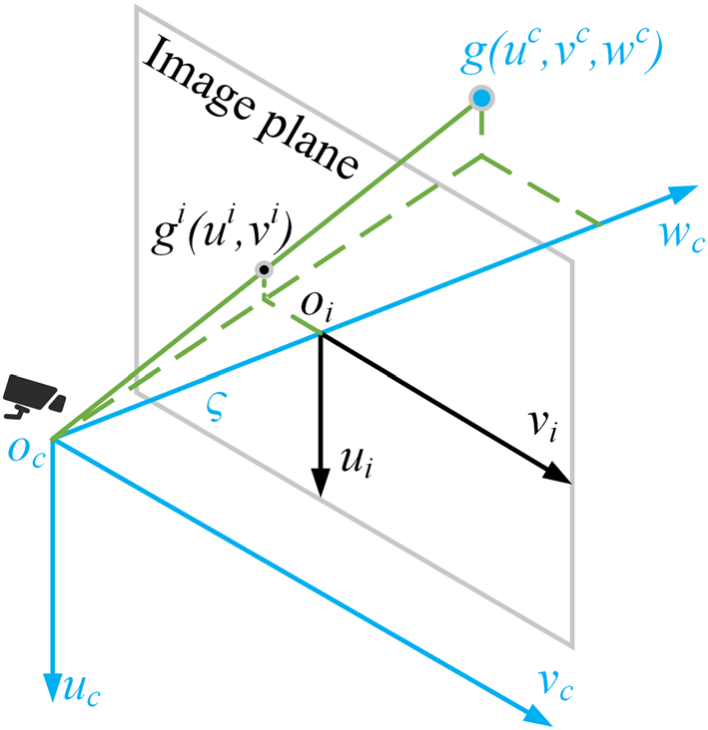
Pinhole camera model.

The camera senses the environment by mapping objects onto the image plane. In traffic DT generation, the inverse process of this mapping is needed. Specifically, the camera model using the pinhole imaging principle is shown in Fig. 2, the camera detects the object from the image plane $x_{i}y_{i}$ and restores the object’s position in the image to the camera coordinate system $x_{c}y_{c}z_{c}$ for obtaining the ground position of the target.

After establishing the image coordinate and the camera coordinate system as shown in Fig. 2, the rule for mapping objects in the physical world to images is^24^

4 $$\begin{aligned} \left[ \begin{array}{c} u^{i} \\ v^{i} \\ 1 \end{array}\right] =\frac{1}{w^{c}} \left[ \begin{array}{lll} \varsigma &{} 0 &{} 0 \\ 0 &{} \varsigma &{} 0 \\ 0 &{} 0 &{} 1 \end{array}\right] \left[ \begin{array}{c} u^{c} \\ v^{c} \\ w^{c} \end{array}\right] , \end{aligned}$$

where $\varsigma$ is the focal length. In the traffic scenario, the targets of interest are moving on the ground, and the mounting height of the camera can be obtained by measurement. Under the assumption that the ground is flat, the $u^{c}$ coordinate of the target can be considered as known, i.e., equal to the mounting height of the camera. In this way, the transformation from the image coordinate system to the camera coordinate system can be derived from Eq. (4) as

5 $$\begin{aligned} \frac{1}{u^{c}} \left[ \begin{array}{c} u^{c} \\ v^{c} \\ w^{c} \end{array}\right] =\frac{1}{u^{i}} \left[ \begin{array}{lll} 1 &{} 0 &{} 0 \\ 0 &{} 1 &{} 0 \\ 0&{} 0 &{} \varsigma \end{array}\right] \left[ \begin{array}{c} u^{i} \\ v^{i} \\ 1 \end{array}\right] . \end{aligned}$$

The target position in the image can be obtained using state of the art image detection methods such as YOLO and Fairmot^25,26^. Then the position of the target on the ground can be obtained in terms of Eq. (5).

## Methods of digital twin generation

### Coordinate systems

**Figure 3:**
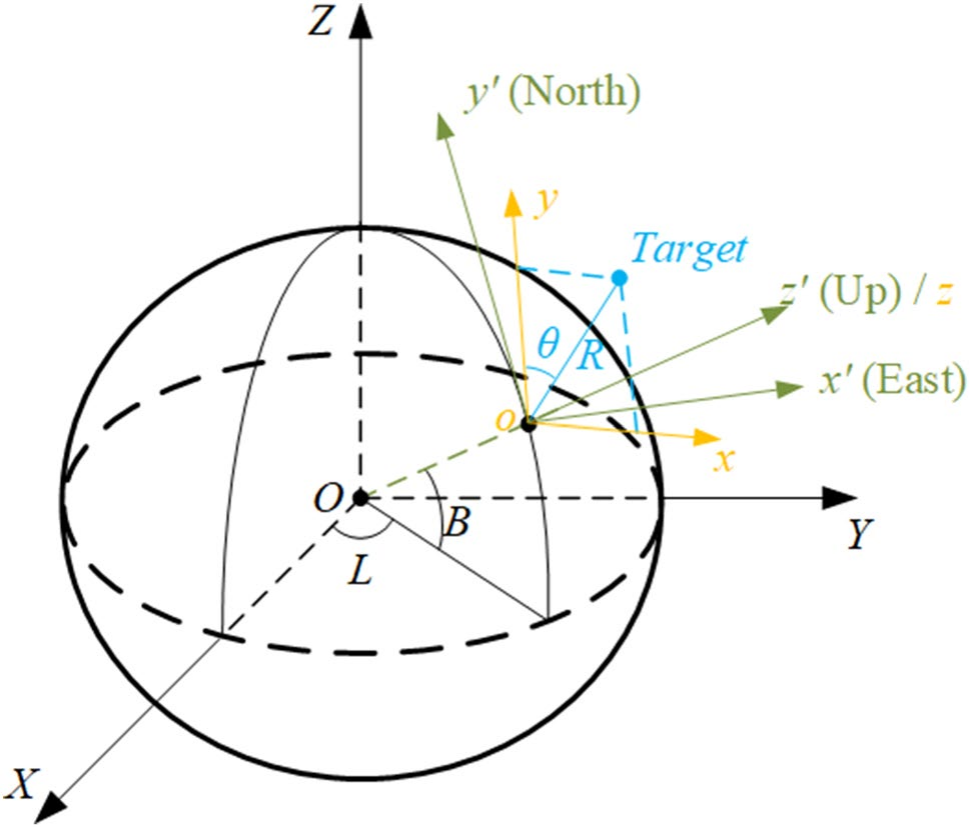
Coordinate systems for DT generation.

In city-scale highway traffic flow sensing applications, a large number of radars and cameras will be deployed at different locations. Since the measurement of target positions by different sensors is usually performed in their own local coordinate systems, these sensors need to be spatially aligned for making the target positions consistent across sensors. A feasible way to alignment of sensors can be realized by choosing a unified coordinate system (UCS)^27,28^. The most common UCS is the WGS-84 system (World Geodetic System)^29^, in which the position of the target is uniquely determined by longitude, latitude, and altitude. When all sensor measurements are converted to WGS-84 system coordinates, spatial alignment can be achieved for all targets in the area covered by these sensors. A typical conversion process from sensor local coordinates system to WGS-84 system is illustrated in Fig. 3. It can be seen from Fig. 3 that the transformation from the sensor local Cartesian (LC) coordinate system, i.e., *xyz*, to WGS-84 system requires the help of intermediate coordinate systems. In this work, the intermediate coordinate systems can be selected as the local east-north-up (ENU) coordinate system, i.e., $x' y' z'$, and Earth-centered Earth-fixed (ECEF) coordinate system, i.e., *XYZ*. It is worth noting that radar sensor measurements of targets are defined in a local polar (LP) coordinate system. In order to convert a target position measured by a radar sensor to WGS-84 system, it is necessary to first convert the LP coordinate to a LC coordinate. According to Fig. 3, the transformation process can be summarized as follows:

1. Conversion of the LP $(R,\theta )$ to LC *xyz* with the same origin. This is for radar sensor only.
2. Conversion of the LC *xyz* to ENU $x' y' z'$ with the same origin and the same *z* axis.
3. Conversion of the ENU $x' y' z'$ to ECEF *XYZ*.
4. Conversion of the ECEF *XYZ* to WGS-84.

**Figure 4:**
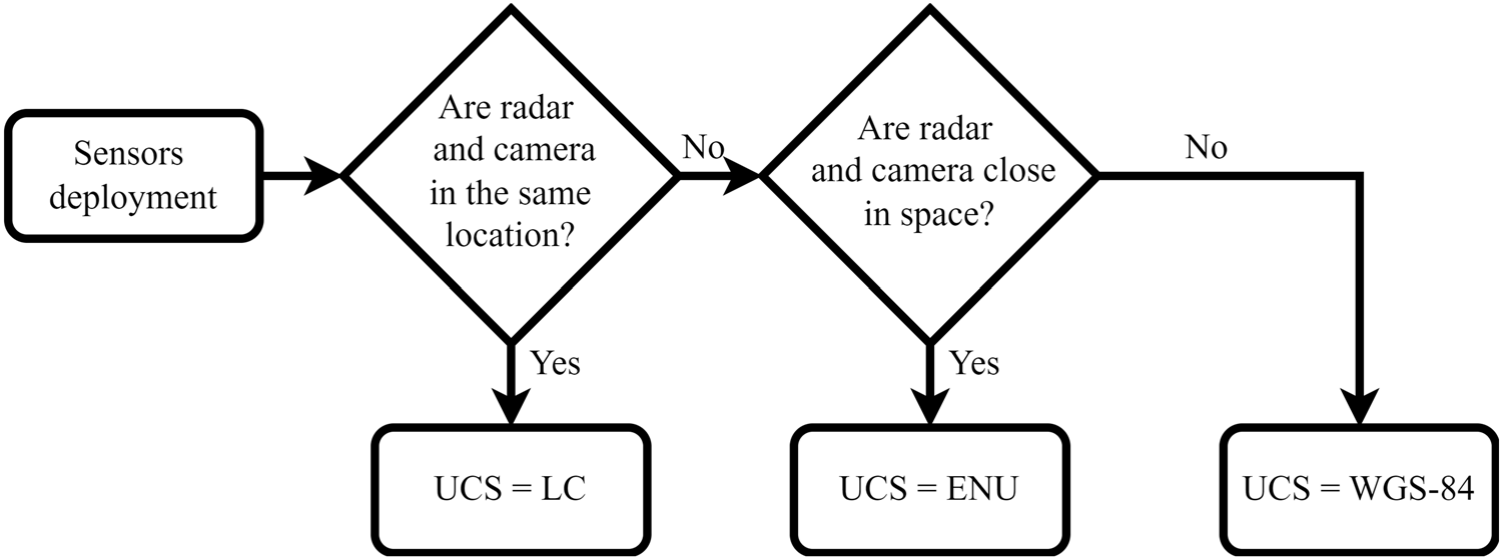
UCS selection in three sensor deployment cases.

Although the use of WGS-84 can solve the sensor alignment problem in arbitrary scenes, it also brings an increase in computational complexity, i.e., each sensor needs to complete the transformation to WGS-84 before post-processing can be performed. In the data fusion application of radar and camera, the choice of UCS can be based on the deployment location of the radar and camera. When the radar and camera are installed close enough, the effect of earth curvature can be neglected and there is no need to select WGS-84 system as UCS. In fact, three coordinate systems can be chosen as UCS depending on the relative deployment positions of the cameras and radars. The details are shown in Fig. 4. For highway sensing, the radar and camera are deployed in pairs at a certain site with the same location. Therefore, LC is adequate to be used as a UCS for a radar-camera paired site.

In this work, radar-camera pairs are used for highway scene sensing, hence LC is chosen as UCS for sensor fusion. LC in this work is defined as follows: the direction normal of the sensor is *y*-axis, pointing to the right of the sensor and perpendicular to the *y*-axis is *x*-axis, pointing above of the sensor and perpendicular to both *x*-axis and *y*-axis is *z*-axis. Based on the radar and camera models, the output of radar measurements are defined in a LP coordinate system, which measures the slant distance *R* and azimuth $\theta$ of the target, and the output of camera measurements are defined in camera coordinate, which measures the two-dimensional (2D) position of the target. In sensors fusion, the radar and camera outputs need to be transformed from their corresponding measurement coordinate systems to the UCS. In this case, the radar LP to LC transformation is defined as

6 $$\begin{aligned} \left\{ \begin{array}{l} x^{r} = R\sin (\theta )\\ y^{r} = R\cos (\theta ) \end{array}\right. . \end{aligned}$$

As shown in Fig. 2, the camera coordinate to LC conversion is defined as

7 $$\begin{aligned} \left\{ \begin{array}{l} x^{c} = v^{c}\\ y^{c} = w^{c} \end{array}\right. . \end{aligned}$$

It is worth noting that since both the radar and the camera measure 2D coordinates of the target in the ground plane, the *z* coordinate in LC is considered as a constant.

**Figure 5:**
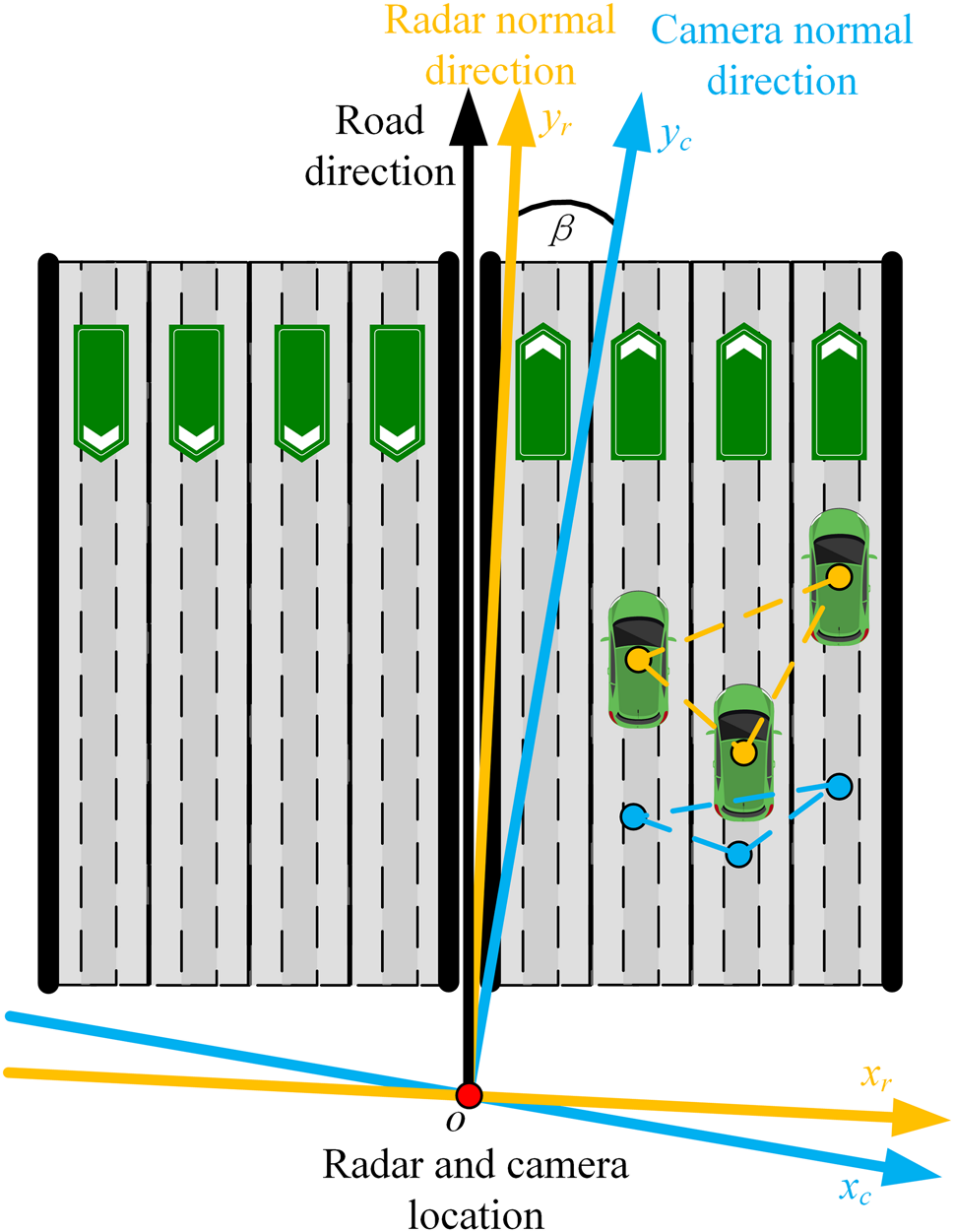
Sensors deployment schematic for radar-camera calibration.

### Adaptive system calibration based on road feature

After determining the UCS, the sensors need to be calibrated before post-processing. The main contents of system calibration is the output data registration of the radar and camera.

In this work, the system calibration of the first case as shown in Fig. 4 is considered, i.e., the radar and the camera are paired in the same location. The schematic diagram of radar and camera normal error is shown in Fig. 5. It can be seen from Fig. 5 that the presence of installation and manufacturing errors make the radar normal and the camera normal not parallel to each other in actual deployment. Due to the error angle $\beta$ between radar and camera normal, the target positions detected by radar and camera cannot appear in the same position even in the same coordinate system. If the radar LC coordinate system is chosen as the UCS, the target position detected by the camera needs to be compensated for the normal error angle before it can be converted to UCS.

When the radar and camera are installed at the same position, there is a rotational transformation relationship between the radar LC and the camera LC due to the angular error between their normal lines. Meanwhile, when the focal length of the camera is unknown, the target positions measured by the camera and the radar have a scale-transformation relationship. Therefore, the relationship between radar and camera LC coordinates is an affine transformation, and there is no translation in the transformation because the origin of their coordinate system overlaps. Hence, the transformation of camera LC to radar LC is given as

8 $$\begin{aligned} \begin{aligned} {\textbf{g}}^{r}&=\textbf{E F g}^{c} \\&=\begin{bmatrix} \rho _{x} &{} 0\\ 0 &{} \rho _{y} \end{bmatrix} \begin{bmatrix} \cos \beta &{} -\sin \beta \\ \sin \beta &{}\cos \beta \end{bmatrix} \begin{bmatrix} x^{c}\\ y^{c} \end{bmatrix}, \end{aligned} \end{aligned}$$

where ${\textbf{E}}$ is the scaling transformation, $\rho _{x}$ and $\rho _{y}$ are the scaling factors of the corresponding coordinate axis respectively, ${\textbf{F}}$ is the rotation transformation, ${\textbf{g}}^{r}=[x^{r},y^{r}]$ and ${\textbf{g}}^{c}=[x^{c},y^{c}]$ is the target coordinates in radar LC and camera LC respectively. The affine transformation in Eq. (8) can be solved using some point cloud registration techniques^30,31^. However, with the help of road features in highway scenario, we can simplify this registration process. In the highway scenario, the intermediate belt is a major straight line feature. If the straight line corresponding to the belt can be localized from the detection results of radar and camera respectively, then the angle between the two straight lines is the angle deviation $\beta$ between the radar normal and the camera normal.

Hough transform is an effective linear detection technique that can be used to detect highway intermediate belt in the results of radar and camera^32,33^. The transform maps a line to the Hough parameter space to accumulate the number of points, where the line can be obtained by threshold detection. The line function defined in Hough transform is

9 $$\begin{aligned} \eta =x \cos (\phi )+y \sin (\phi ) , \end{aligned}$$

where the coordinate (*x*, *y*) is used to describe the target position for sensors, while each point $(\eta , \phi )$ in Hough parameter space represents a line in the input 2D position matrix. The score of corresponding point in the parameter space can be measured as

10 $$\begin{aligned} \begin{aligned} H(\eta , \phi )&=\iint _{L} \delta (x, y) d x d y , \\ \text {with } \delta (x, y)&=\left\{ \begin{array}{l}1, \text{ if } (x, y) \text{ is } \text{ on } L \\ 0, \text{ otherwise } \end{array}\right. , \end{aligned} \end{aligned}$$

where *L* denotes that the line satisfies with Eq. (9). After obtaining all the scores of parameter space, lines can be extracted if $H_{p}(\eta , \phi )$ is greater than a specified threshold, and line position in input matrix is

11 $$\begin{aligned} \left\{ \begin{array}{llrl} x &{} =\eta , &{} &{} \text{ if } \sin (\phi )=0 \\ y &{} =-\cot (\phi ) x+\frac{\eta }{\sin (\phi )}, &{} &{} \text{ otherwise } \end{array}\right. . \end{aligned}$$

It is worth noting that the input matrix can be either target positions detected by the radar or an image recorded by the camera. After obtaining the intermediate belt straight line detected by the radar, i.e., $\vec {l}^{r}$, and the camera, i.e., $\vec {l}^{c}$, respectively, the angle $\beta$ between the two straight lines can be calculated as

12 $$\begin{aligned} \beta =\arccos \left( \frac{\left| \vec {l}^{r} \cdot \vec {l}^{c}\right| }{\left| \vec {l}^{r}\right| \left| \vec {l}^{c}\right| }\right) , \quad \beta \in \left[ 0^{o}, 90^{\circ }\right] , \end{aligned}$$

and the rotation transformation can be obtained in terms of Eq. (8), then the affine transformation defined in Eq. (8) is simplified as

13 $$\begin{aligned} \begin{aligned} {\textbf{g}}^{r}&=\textbf{E g}^{c}_{F} \\&=\begin{bmatrix} \rho _{x} &{} 0\\ 0 &{} \rho _{y} \end{bmatrix} \begin{bmatrix} x^{c}_{F}\\ y^{c}_{F} \end{bmatrix}. \end{aligned} \end{aligned}$$

In this case, the remaining calibration work is to estimate the scaling transformation matrix ${\textbf{E}}$. Some vehicle targets in the highway scenario can be selected as feature points. For instance, the detected positions for three vehicles by radar and camera form two triangles respectively as shown in Fig. 5a. The relationship between these two triangles is scale-transformed, and the scaling transformation can be derived in terms of Eq. (13) as

14 $$\begin{aligned} \begin{aligned} {\textbf{E}}&= [{\textbf{G}}^{r}({\textbf{G}}^{c}_{F}) ^{*}] [{\textbf{G}}^{c}_{F}({\textbf{G}}^{c}_{F})^{*}]^{-1},\\ \text {with } {\textbf{G}}^{r}&=({\textbf{g}}^{r_{1}},{\textbf{g}}^{r_{2}},...,{\textbf{g}}^{r_{N}})\\ {\textbf{G}}^{c}_{F}&=({\textbf{g}}^{c_{1}}_{F},{\textbf{g}}^{c_{2}}_{F},...,{\textbf{g}}^{c_{N}}_{F}), \end{aligned} \end{aligned}$$

where $*$ denotes matrix transposition. It is worth noting that the target number should satisfy $N\ge 2$ to ensure that $[{\textbf{G}}^{c}_{F}({\textbf{G}}^{c}_{F})^{*}]^{-1}$ exists.

After the scaling transformation ${\textbf{E}}$ and rotation transformation ${\textbf{F}}$ are obtained, the conversion from camera LC coordinates to radar LC coordinates can be realized in terms of Eq. (8).

**Figure 6:**
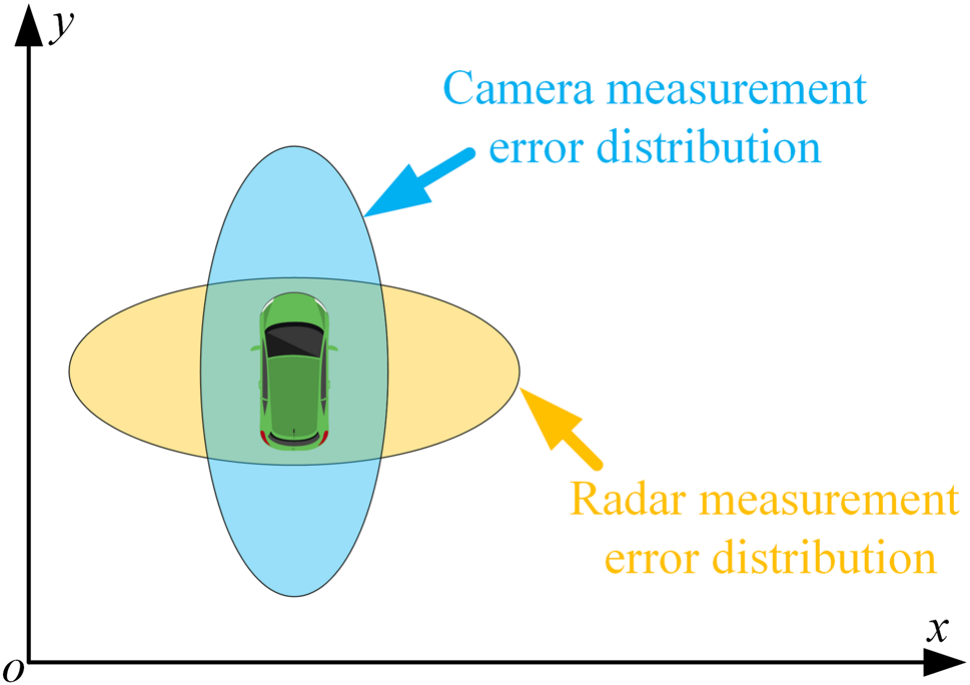
Measurements error distribution for different sensors.

### Radar-camera fusion detection and tracking

According to sensor characteristics, radar is more accurate in distance and velocity measurements, while camera is more accurate in angle, height and target class measurements^34^. The measurement accuracy of radar and camera usually has the distribution as shown in Fig. 6. Based on their respective advantages in target measurement, a novel radar-camera fusion framework is proposed in this section.

After the target positions detected by the radar and camera are converted to the UCS, the targets tracking based on sensors fusion can be realized. Since the goal is to obtain the target trajectory after sensor fusion, the Kalman filter (KF) framework is adopted in this paper for achieving both fusion and subsequent tracking^35,36^. For radar-camera fusion in traffic applications, the target dynamics and measurements of sensors can be modeled as a system which has the same state equation and multiple measurement equations

15 $$\begin{aligned} {\textbf{u}}_{k}= & {} {\textbf{A}} {\textbf{u}}_{k-1} + \mathbf {\varepsilon }, \end{aligned}$$

16 $$\begin{aligned} {\textbf{g}}_{k}^{r}= & {} {\textbf{C}} {\textbf{u}}_{k} + \mathbf {\zeta }^{r}, \nonumber \\ {\textbf{g}}_{k}^{c}= & {} {\textbf{C}} {\textbf{u}}_{k} + \mathbf {\zeta }^{c}, \end{aligned}$$

where *k* is the discrete time, ${\textbf{A}}$ is the state transfer matrix, ${\textbf{u}}_{k}$ is the state vector, i.e., the target position determined by the target motion equation, ${\textbf{g}}_{k}^{r}$ and ${\textbf{g}}_{k}^{c}$ is the measurement vector of radar and camera respectively, $\mathbf {\varepsilon }$ and $\mathbf {\zeta }$ are the process noise and measurement noise with covariance matrices $\mathbf {\aleph }$ and $\mathbf {\Re }$ respectively. It is worth noting that the state-to-measurement matrix ${\textbf{C}}$ is equal to identity matrix, since both the state space and measurement space are in the UCS. Sensors fusion can be achieved by either state vector fusion or measurement fusion, and the latter one has been shown to provide better performance^35,36^. In this paper, the measurements of the radar and camera are combined for establishing a target tracking framework which is suitable for traffic scenarios.

**Figure 7:**
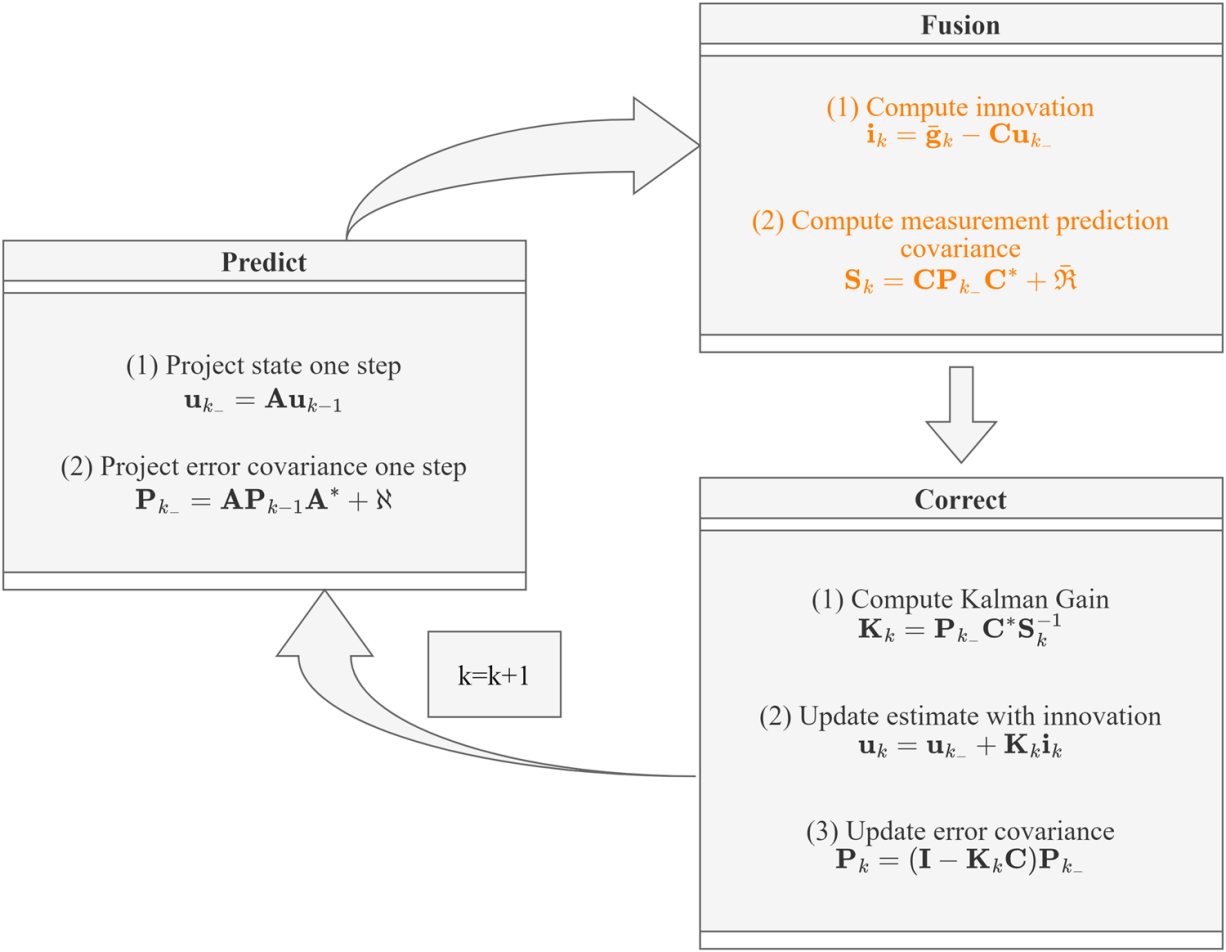
Fusion Kalman filter framework.

In traffic sensing application, as shown in Fig. 6, the measurement of the radar has a large variance component level in the *x*-axis, while the measurement of the camera has a large variance component level in the *y*-axis. After setting the measurement covariance matrix $\mathbf {\Re }^{r}$ and $\mathbf {\Re }^{c}$ for radar and camera according to the measurement error distribution characteristics, sensor information fusion is achieved in two parts:

1. The target measurement positions fusion of radar and camera, which is computed as
  17 $$\begin{aligned} \bar{{\textbf{g}}}_{k}={\textbf{g}}_{k}^{r}+\mathbf {\Re }^{r}\left( \mathbf {\Re }^{r}+\mathbf {\Re }^{c}\right) ^{-1}\left( {\textbf{g}}_{k}^{c}-{\textbf{g}}_{k}^{r}\right) . \end{aligned}$$
2. The fusion of radar and camera measurement errors, which is computed as
  18 $$\begin{aligned} \bar{\mathbf {\Re }}=\left[ \left( \mathbf {\Re }^{r}\right) ^{-1}+\left( \mathbf {\Re }^{c}\right) ^{-1}\right] ^{-1}. \end{aligned}$$

With the fusion results of measurement $\bar{{\textbf{g}}}_{k}$ and measurement covariance $\bar{\mathbf {\Re }}$, denote ${\textbf{I}}$ as the identity matrix, the implementation flow of the fusion tracking approach is shown in Fig. 7.

### DT generation approach for highway scenario

**Figure 8:**
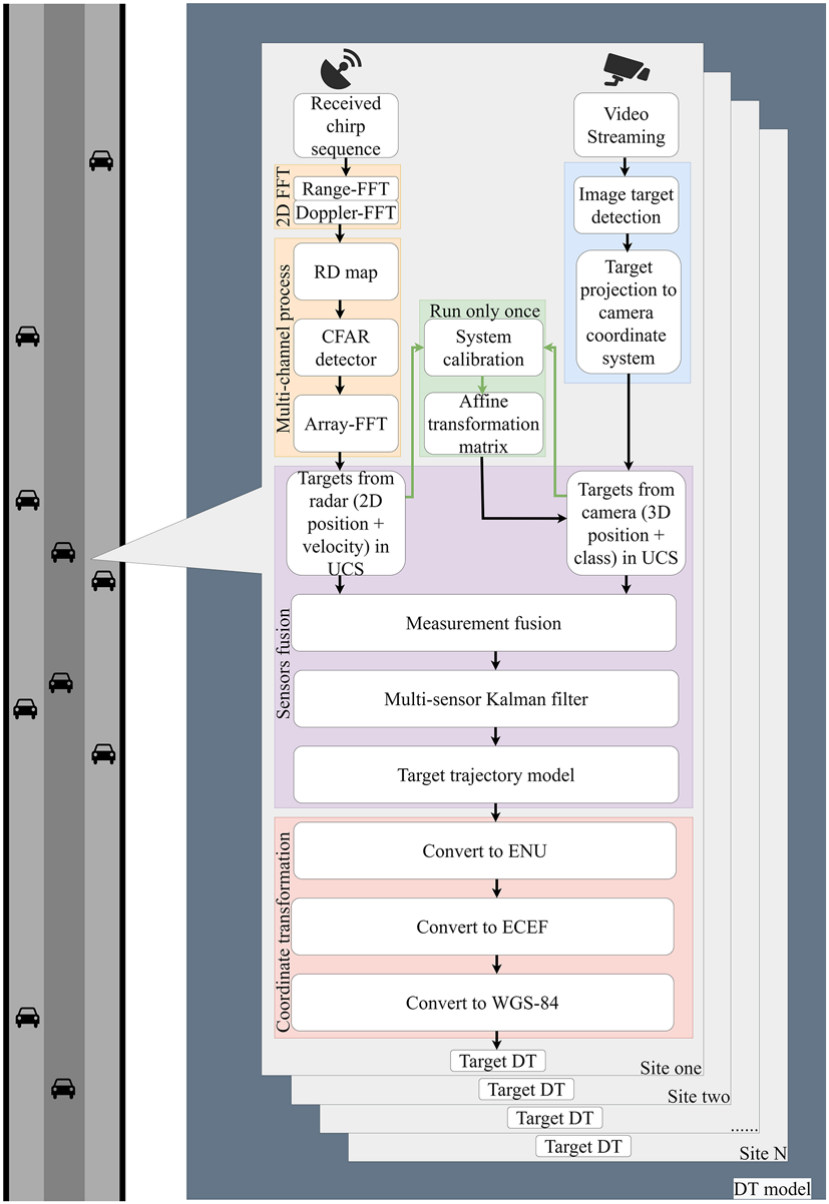
End to end DT generation approach.

In a nutshell, the approach of DT model generation is shown in Fig. 8. For highway scenario, radar-camera pairs can be deployed at multiple sites along the road, and each pair of radar and camera is responsible for traffic flow sensing in a local area. For a single site, the radar and the camera acquire their respective sensory data. The radar obtains 2D position and velocity information of the target after signal processing. The camera obtains the 3D position information of the target by image processing followed by a image plane to UCS conversion. In UCS, the detection data of the two sensors are fused and the trajectory of the target is obtained by the fusion Kalman filter as shown in Fig. 7, which completes the generation of local traffic flow DT.

When the traffic flow data of all sites are obtained, the traffic flow information of each site is converted to WGS-84 system by coordinate conversion, and the DT model of the whole highway scenario can be generated.

Here are some factors to consider in DT model generation:

1. Besides detecting the 2D position of the target, the radar can be used to measure the target velocity more accurately. In practice, the radar velocity information can be output as needed.
2. The camera provides a more accurate measurement of the width, height, and class of the target, besides detecting the 2D position of the target. These information can be output in practice as attached attributes based on demand.
3. After DT model generation, target locations in the DT model need to be exported in practical applications. Similar to the coordinate system considered for sensor fusion, the model output also requires the selection of coordinate system according to practical applications. The presentation of the DT model in WGS-84 coordinate system is not required. It depends on whether a large-scale scene needs to be modeled and whether that DT model needs to be fused with the map system. When fusion with the map system is required, traffic target information needs to be transformed to WGS-84. When fusion with the map system is not required, for single-site models, the target information can be output directly in the LC. For small-scale models, such as several intersections, transformation to ENU coordinate system is sufficient, and for large-scale models, such as city level and above, transformation to ECEF is sufficient.

## Experimental results and discussion

### Data collection and performance evaluation metrics

**Figure 9:**
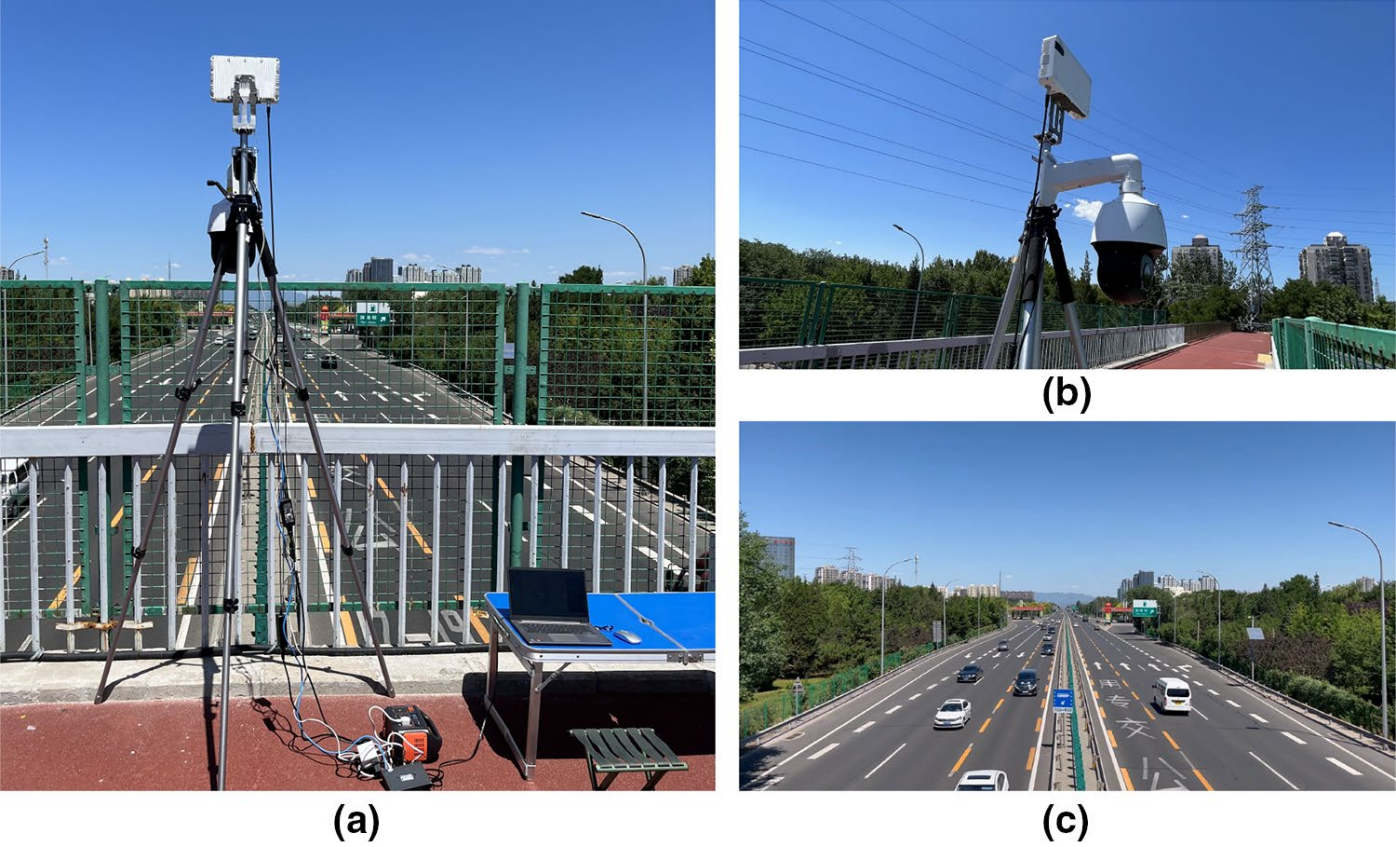
Experimental scenario. (**a**) Data acquisition equipment configuration, (**b**) Sensors deployment, (**c**) Highway scenario.

We tested the proposed DT model generation approach based on a real highway scenario. In the experiment, a traffic radar and a camera were used for data collection. The experiment was located on an overpass on the highway, and the radar and camera were installed at the same location with a height of 8 m from the ground as shown in Fig. 9a,b respectively. The highway in the experiment scenario is a bidirectional six-lane road with a separation zone in the middle of the road, as shown in Fig. 9c. The technical parameters of the radar and camera are shown in Tables 1 and 2 respectively.

**Table 1:** The radar system parameters.

Parameter name	Value
Operating frequency (GHz)	24
Ranging accuracy (m)	0.8
Speed measuring accuracy (m/s)	0.03
Angle measuring accuracy (°)	0.4
Horizontal field of view (°)	45
Vertical field of view (°)	29

**Table 2:** The camera system parameters.

Parameter name	Value
Image resolution	1920 × 1080
Focal length (mm)	15
Horizontal field of view (°)	8.1
Vertical field of view (°)	4.5

We employ the multiple object tracking accuracy (MOTA) metric^37^, which is commonly used in multi-target tracking, to measure the performance of fusion tracking. The MOTA in time *t* is defined as

19 $$\begin{aligned} \textrm{MOTA}=1-\frac{\sum _{t}\left( MI_{t}+F P_{t}+M M E_{t}\right) }{\sum _{t} GT_{t}}, \end{aligned}$$

where $MI_{t}$, $F P_{t}$, and $M M E_{t}$ are the number of misses, of false positives, and of mismatches, respectively. $GT_{t}$ is the number of objects in the scene. From Eq. (19), The MOTA can be seen as derived from 3 error ratios, i.e., the ratio of misses (ROM),

20 $$\begin{aligned} ROM=\frac{\sum _{t} MI_{t}}{\sum _{t} GT_{t}}, \end{aligned}$$

the ratio of false positives (ROFP),

21 $$\begin{aligned} ROFP=\frac{\sum _{t} F P_{t}}{\sum _{t} GT_{t}}, \end{aligned}$$

and the ratio of mismatches (ROMM),

22 $$\begin{aligned} ROMM=\frac{\sum _{t} M M E_{t}}{\sum _{t} GT_{t}}. \end{aligned}$$

In the experiment, the target is detected by the radar and camera separately. The radar obtains the target’s position information by transmitting FMCW waveform, performing FFTs processing on the target echoes and detecting the target by CFAR. The camera uses Fairmot to detect the position of the target in the video. After getting the detection results from the radar and camera, the proposed fusion Kalman filter is used to track multiple targets in the scene during the generation of the traffic flow DT. The tracked trajectories are counted to obtain quantitative MOTA results, while the results for the intermediate metrics ROM, ROFP, and ROMM are obtained too. In the performance results, tracking results of radar only and camera only are also given for comparison. Meanwhile, two fusion tracking strategies, i.e., heuristic fusion with adaptive gating (HFAG)^20^ and track-to-track fusion (TTF)^19^, are employed, and compared with the detection-to-detection fusion proposed in this paper.

### Results and discussion

**Figure 10:**
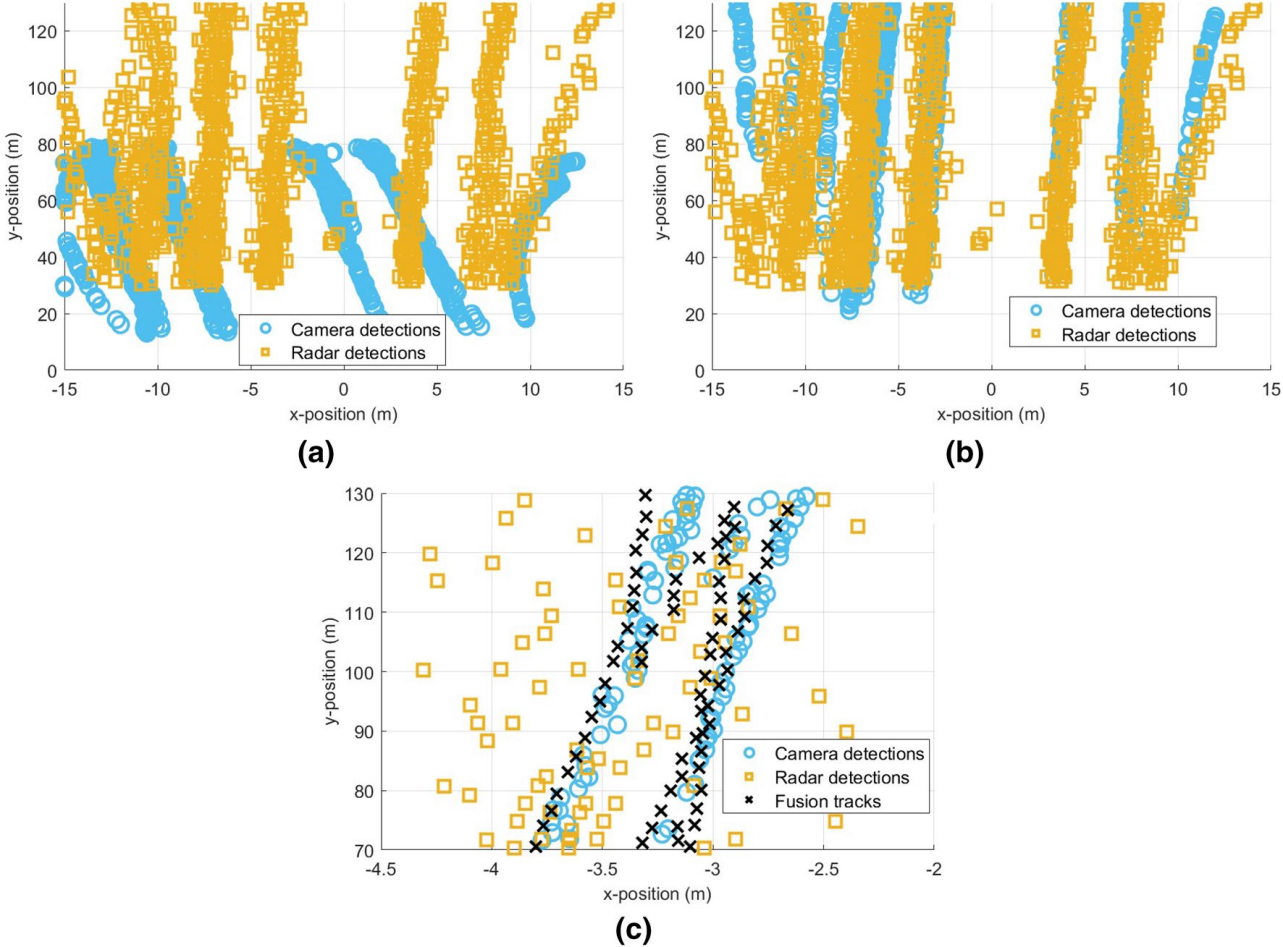
Radar-camera calibration. (**a**) Detections of car flows before sensor calibration in radar and camera LCs, the axes of the two LC systems are overlapped and drawn together, (**b**) Detections of car flows after sensor calibration in UCS, (**c**) Car tracks outputed by fusion Kalman filter after sensor calibration in zoomed-in UCS.

Radar echo signals and camera video were recorded simultaneously during the experiment. The target positions measured by radar and camera are converted to their respective LC systems and the car flow detections are drawn in Fig. 10a. It can be seen that the car flow detections are not aligned before calibration due to the spatial errors between the two sensors. Specifically, the directions of car flows from different sensors are not the same, and the scales in the *y*-direction are also inconsistent. Taking the radar LC system as UCS, the calibration results of the car flow detections are shown in Fig. 10b. It can be seen that the normal error and the scale inconsistency between the two sensors have been corrected, and the car flow directions are kept consistent. In this case, the position difference between the detection points of radar and camera is mainly caused by two factors. The first factor is the calibration residual, which is reflected in the overall deviation of the detections between the radar and camera as shown in Fig. 10b. The second factor is the measurement error of the sensors themselves. From the zoomed-in plot as shown in Fig. 10c, the x-position error of the radar detections is about 1m, and the x-position error of the camera detections is about 0.2 m. After the target tracking by the fusion Kalman filter, the position error is further eliminated and accurate target position information can be obtained.

It is worth noting that it is necessary to choose proper measurement accuracy sensors according to the application requirements in practice. For example, we want to obtain a DT model with lane level accuracy in this experiment, hence the measurement error of the selected sensor at x-position, usually for radar, should be less than 3 m, considering that the width of the lane is usually larger than 3 m.

The performance of target tracking is evaluated using the measured traffic flow data of the highway scenario. To quantitatively evaluate the MOTA metrics, we manually labeled 1000 frames of data in the experiment, and the trajectory output results of the tested methods were labeled with missed detection, false positives and mismatches. The quantitative results of the target tracking in the DT model are shown in Table 3. It can be seen that the performance of target tracking is improved by sensor fusion with respect to either radar only or camera only method. Among the three tested fusion methods, the HFAG method uses a predefined correlation gate, and if the scene does not match the predefined gate in practice, the tracking performance will be degraded. Compared with the HFAG method, the track-to-track fusion and the proposed method obtain better performance, however, the track-to-track fusion requires at least 3 Kalman filtering processes, i.e., the respective Kalman filtering of radar and camera, and a fusion Kalman filtering, which increases the computational burden in practice.

**Table 3:** The tracking performance of different methods.

Target tracking methods	ROM (%)	ROFP (%)	ROMM (%)	MOTA (%)
Radar only	8.7	0.7	2.7	87.9
Camera only	3.3	2.1	0.8	93.8
HFAG^20^	3.1	1.1	0.7	95.1
TTF^19^	2.7	0.63	0.66%	96.0
The proposed method	2.3	0.61	0.71	96.38

A DT model is generated for the highway scenario using the fusion tracking results of the car flow from radar and camera. The digitized car flow locations are mapped to the WGS-84 coordinate system, then the DT model is displayed by using the WGS-84 coordinates on a satellite map corresponding to the experimental site provided by AutoNavi as shown in Fig. 11a. The yellow rectangle represents the vehicle target, and the solid yellow dot on the rectangle represents the vehicle front facing. The scenario video of the same moment is also given in Fig. 11b. The corresponding cars are marked by red numbers in both Fig. 11a,b, it can be seen that the relative positions between cars in the DT model are correctly reflected compare with the scenario video. Meanwhile, the DT projection on the satellite map are correct and accurate, since the cars all appeared in the correct lanes. In addition, there are several details worth noting here:

1. As can be seen in the video shown in Fig. 11b, Car 1 is changing lanes at this moment, and as can be seen in the DT model on the satellite map shown in Fig. 11a, Car 1 is also on the dashed line on the ground between the two lanes.

**Figure 11:**
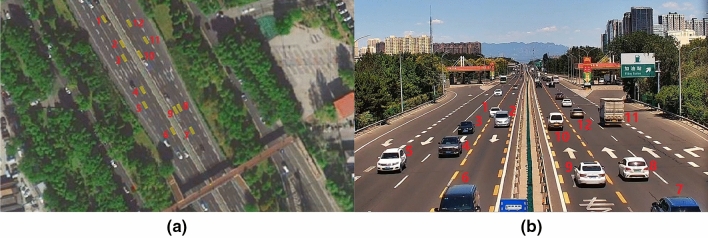
DT model of the experimental highway scenario. (**a**) Car flow DT in satellite map, (**b**) Scenario video in the same moment.
2. In the video, Car 3 travels to the end of the ground arrow marker, and in the satellite map, the DT model of Car 3 travels to the same spot of the ground arrow marker.
3. In the video, Car 8 and Car 9 are driving side-by-side in adjacent lanes, with Car 9 slightly behind Car 8 by about half a body length, and this position relationship between Car 8 and Car 9 is reflected by their DT models in the satellite map. It is worth noting that this side-by-side traffic status is a challenging corner case for radar tracking due to the low angle resolution, which can be successfully solved by fusion with camera.

## Conclusion

In this paper, an end-to-end generation approach for DT model based on radar and camera fusion is proposed for highway scenario. Starting from the raw data of sensors, the deployment error of the sensing system is calibrated using the road feature information to make the radar and camera pointing consistent. After sensor calibration, the data from different sensors are transformed into a UCS and the targets are tracked in this coordinate system using fusion Kalman filter to obtain accurate motion trajectory. As a result, a DT model of the traffic flow is built. Finally, the DT model can be optionally transformed to the desired coordinate system for post-processing. The effectiveness of the proposed method is verified by building a DT model of the traffic flow in a real highway scenario.

Using the DT model built for the highway scenario, road conditions can be dynamically captured in real time and extrapolated for the real physical world state. Based on this prediction information the traffic efficiency of the road can be optimized, and further, the DT model can be iterated by the real physical world situation. So on and so forth, using the interaction of digital and physical twins will effectively enhance the functionality of the intelligent transportation system. In the above application, the method proposed in this paper is a candidate for obtaining the DT model of traffic flow.

Based on the progress of the current work, the following directions for subsequent research are available:

1. When there is target missing from any sensor in the radar-camera pair, how to deal with such target and improve the tracking accuracy is the work that needs to be continued on the basis of this paper.
2. Limited by the current experimental conditions, we are unable to build a digital model for a larger scale scenario, which will be the focus of our subsequent work.
3. When the information of multiple sites is connected to form a DT model of a large scene, the target association between sites will be a complex problem if there is a coverage overlap area between the sensors of each site, and the handling of this problem will determine the accuracy of the DT model, which is the direction of subsequent research.

## Data Availability

The datasets used and/or analysed during the current study available from the corresponding author on reasonable request.
